# Polymorphisms CYP2R1 rs10766197 and CYP27B1 rs10877012 in Multiple Sclerosis: A Case-Control Study

**DOI:** 10.1155/2021/7523997

**Published:** 2021-12-23

**Authors:** A. Martinez-Hernandez, E. E. Perez-Guerrero, M. A. Macias-Islas, C. A. Nava-Valdivia, A. Villagomez-Vega, B. Contreras-Haro, Y. E. Garcia-Ortega, Y. Esparza-Guerrero, S. G. Gallardo-Moya, J. I. Gamez-Nava, L. Gonzalez-Lopez, E. Oliva-Flores, N. A. Rodriguez-Jimenez, F. Cortes-Enriquez, A. M. Saldaña-Cruz

**Affiliations:** ^1^Programa de Doctorado en Farmacología, Departamento de Fisiología, Centro Universitario de Ciencias de la Salud, Universidad de Guadalajara, Guadalajara, Mexico; ^2^Instituto de Investigación en Ciencias Biomédicas, Centro Universitario de Ciencias de la Salud, Universidad de Guadalajara, Guadalajara, Jalisco, Mexico; ^3^Departamento de Neurociencias, Centro Universitario de Ciencias de la Salud, Universidad de Guadalajara, Guadalajara, Jalisco, Mexico; ^4^Departamento de Microbiología Y Patología, Centro Universitario de Ciencias de la Salud, Universidad de Guadalajara, Guadalajara, Jalisco, Mexico; ^5^Departamento de Ciencias Biomédicas, Centro Universitario de Tonalá, Universidad de Guadalajara, Tonalá, Mexico; ^6^Hospital de Especialidades, Centro Médico Nacional de Occidente, Guadalajara, Jalisco, Mexico; ^7^Doctorado en Salud Pública, Departamento de Salud Pública, Centro Universitario de Ciencias de la Salud, Universidad de Guadalajara, Guadalajara, Jalisco, Mexico; ^8^Instituto de Terapéutica Experimental Y Clínica, Departamento de Fisiología, Centro Universitario de Ciencias de la Salud, Universidad de Guadalajara, Guadalajara, Jalisco, Mexico; ^9^Instituto Mexicano del Seguro Social, Guadalajara, Jalisco, Mexico

## Abstract

**Background:**

Multiple sclerosis (MS) is a chronic autoimmune inflammatory disease. Low vitamin D levels have been reported to be a risk factor for MS, and genetic variances could be implicated. The aim of this study was to evaluate the association of MS with rs10766197 polymorphism of *CYP2R1* gene and rs10877012 polymorphism of *CYP27B1* gene. The second aim was to analyse whether these polymorphisms are associated with the severity of the progression of MS. *Material and Methods*. In a case-control study, we included 116 MS patients and 226 controls, all of whom were Mexican Mestizo. MS was diagnosed by McDonald criteria (2017). A complete neurological evaluation was performed to evaluate the severity of disease progression. Serum 25-hydroxyvitamin D [25(OH) vitamin D] levels were measured by ELISA. Single nucleotide polymorphisms rs10766197 of *CYP2R1* gene and rs10877012 SNP of *CYP27B1* gene were genotyped by real-time PCR.

**Results:**

Serum 25(OH) vitamin D levels were lower in MS patients than in controls (*p* = 0.009). No differences were observed between serum 25(OH) vitamin D levels of MS patients with severe progression compared to low progression (*p* = 0.88). A higher frequency of the A allele of *CYP2R1* rs10766197 was observed between MS patients and controls (*p* = 0.05). No differences were observed in the frequency of T allele of *CYP27B1* rs10877012 (*p* = 0.65). In subanalysis, patients with GA + AA genotypes of *CYP2R1* rs10766197 had an increased risk of MS compared to controls (*p* = 0.03). No increased risk was observed in GT + TT genotypes of *CYP27B1* rs10877012 (*p* = 0.63). No differences were observed in allele frequencies of either polymorphism between patients with severe vs. low disease progression.

**Conclusion:**

Lower serum 25(OH) vitamin D levels were observed in MS patients than in controls, although these levels were not associated with disease progression. Carriers of GA + AA genotypes of *CYP2R1* rs10766197 had an increased risk of MS. None of these polymorphisms was associated with severe progression of MS.

## 1. Introduction

Multiple sclerosis (MS) is a chronic inflammatory demyelinating disease of the central nervous system (CNS) [1]. Typically, MS affects the brain, spinal cord, and optic nerves, with inflammatory lesions derived from probable autoimmune mechanisms; these lesions are followed by axonal and grey-matter neurodegenerative processes associated with progressive worsening of disability. Most patients experience their first symptoms between the ages of 20 and 40 years. MS is considered the leading cause of disability in young people, excluding injuries caused by traffic accidents [2]. In Mexico, the prevalence of MS is 12 to 15 per 100,000 inhabitants [3].

There are four categories of MS: clinically isolated syndrome (CIS), relapsing-remitting (RRMS), secondary progressive (SPMS), and primary progressive (PPMS), with RRMS accounting for 85% of patients [4]. The World Health Organization (WHO) estimates that more than two million people worldwide suffer from MS [5].

Clinical symptoms of MS may include motor dysfunction, tremor, dysmetria, cerebellar ataxia, nystagmus, diplopia, hypoesthesia, blindness (usually unilateral), and sphincter disturbances such as retention or incontinence; approximately 45 to 60% of patients will develop cognitive decline and neuropsychiatric symptoms [6]. Motor disorders are the most common symptoms in the course of the disease which lead to motor disability [7].

MS is currently considered a very heterogeneous disorder in which both genetic susceptibility and environmental exposures are strongly implicated in T cell activation and pathogenesis [8]. Epstein-Barr virus, cytomegalovirus, and mononucleosis infections; smoking; adolescent obesity; night work; low sun exposure; and 25-hydroxyvitamin D (25(OH) vitamin D) < 30 ng/mL are recognized as risk factors for MS [9]. Vitamin D has been proposed as a serum biomarker in several clinical conditions, including neurodegenerative disorders; additionally, several inflammatory and cardiovascular markers reflect the immunomodulatory function of vitamin D [10]. It has been hypothesized that lower 25(OH) vitamin D levels are associated with the risk of MS [9, 11]. Possible explanations for the association between MS risk and altered vitamin D status alterations include the role of vitamin D in CD4+ T cell balance and remyelination processes, B lymphocyte class switching, immunoglobulin production, and proinflammatory interleukin secretion [12, 13] as well as the presence of vitamin D-responsive elements (VDREs) in the promoter regions of various MS-associated genes [14].

The metabolism of vitamin D, or cholecalciferol, is carried out by enzymes of the cytochrome p450 family, which catalyse a series of hydroxylation reactions to produce different active compounds [15]. CYP2R1 is responsible for the primary hepatic metabolism of vitamin D to 25(OH) vitamin D, which is subsequently converted to 1,25-dihydroxyvitamin D, the primary bioactive vitamin D metabolite, by CYP27B1in the kidney [15–17]. By binding to the vitamin D receptor, a member of the nuclear receptor superfamily, this metabolite mediates functions in multiple classes of cells, including immune cells [18].

Therefore, it is of great importance to study genetic factors that may modify 25(OH) vitamin D levels and have consequential impacts on the disease. The *CYP2R1* gene, found on chromosome 11p15.2, encodes the enzyme 25-hydroxylase, which is involved in the first step of vitamin D hydroxylation activation. The rs10766197 polymorphism of the *CYP2R1* gene has been shown to be an important factor with a major impact on vitamin D levels [19, 20]. The *CYP27B1* gene is located on chromosome 12q13.1-q13.3 and encodes the enzyme 1-alpha-hydroxylase. The rs10877012 polymorphism has been associated with reduced vitamin D levels; thus, together with the rs10766197 polymorphism, it is a promising object of research due to its potential influence in patients with MS (Ramos 2008) [20].

Prior research has found an association the rs10766197 SNP of the *CYP2R1* gene and the risk of MS as well as the progression of the disease [19]; but there has been no prior research on the rs10877012 SNP of the *CYP27B1* gene in patients with MS.

Therefore, the first aim of this study was to evaluate the association of MS with the rs10766197 polymorphism of the *CYP2R1* gene and the rs10877012 polymorphism of the *CYP27B1* gene. The second aim was to analyse whether these polymorphisms are associated with the severity of MS progression.

## 2. Materials and Methods

### 2.1. Study Design

The design of this study is a Case-Control Study, in which the cases were patients with RRMS and the controls were healthy patients without MS, residents of the Guadalajara metropolitan area.

### 2.2. Clinical Setting

This study included 116 RRMS patients and 226 controls. The MS cases were patients treated at the Mexican Association for Multiple Sclerosis A.C. in Guadalajara, Mexico, from January 24, 2019, to August 31, 2021.

### 2.3. Study Subjects

All individuals were aged ≥ 18 years and were Mexican-Mestizo as defined by the Mexican National Institute of Anthropology and History: “individuals who were born in Mexico, of the original autochthonous inhabitants of the region and individuals who were mainly Spaniards” [21].

Controls were selected randomly from the population available at the Clinical and Experimental Therapeutic Institute, consisting of 185 women and 41 men. Controls were included if they had no history of inflammatory or autoimmune disorders, and only one person per family was recruited. Controls were included to compare serum 25(OH) vitamin D levels and the genotypes of the polymorphisms.

### 2.4. Clinical Assessments

Patients with RRMS were included and assessed by an experienced neurologist and based on a previous history of disease and physical examination. Complete neurological examination assured that the patients fulfilled the 2017 McDonald clinical criteria. We excluded patients with kidney disease, liver disease, other uncontrolled autoimmune or psychiatric diseases, and current pregnancy.

Functional systems assessment was used to establish the disability score according to Kurtzke's Extended Disability Status Scale (EDSS) [22]. To ensure a correct diagnosis, we performed 1.5-tesla MRI with conventional T1, gadolinium-enhanced T1, T2, and fluid-attenuated inversion recovery (FLAIR) sequences and confirmed the presence of typical round hyperintense lesions in T2 and FLAIR sequences and hypointensity in T1 with or without enhancement distributed in a different location; the time of evolution differed for each patient [23]. Oligoclonal bands were not considered mandatory to support or exclude an MS diagnosis [24]. Disability was measured using EDSS scores [22]. Disease progression was assessed using the progression index (PI). This index was calculated by dividing the degree of disability, as measured using the EDSS, by the duration of the disease; the typical PI value is 0.4-0.6 [25]. We used the upper end of this range as a cut-off point to dichotomize MS patients into two groups: PI > 0.6 (severe progression) and PI ≤ 0.6 (low progression).

Two experienced and trained researchers recorded the clinical and epidemiological characteristics of the participants, which included age, sex, treatment, duration of MS, and PI.

### 2.5. Genotyping *CYP2R1* rs10766197 and *CYP27B1* rs10877012 Polymorphisms

Peripheral whole blood samples, harvested in Vacutainer tubes containing ethylenediaminetetraacetic acid as an anticoagulant, were collected from each patient. Genomic DNA was obtained from these blood samples using the modified Miller technique [26]. Genomic DNA was quantified using a Nanodrop Genomic, and the DNA was diluted in Tris-EDTA buffer to 20 ng/*μ*L and placed in 200 *μ*L propylene cryotubes (Eppendorf™). Genotyping of *CYP2R1* rs10766197 and *CYP27B1* rs10877012 polymorphisms was performed by quantitative polymerase chain reaction (qPCR) using TaqMan probes [27]. TaqMan Assay IDs C_2958435_10 and C_26237740_10 were performed according to the manufacturer's instructions (Applied Biosystems); the StepOne™, Real-Time polymerase chain reaction (qPCR) system was employed for this purpose (Applied Biosystems). All results were independently scored by two investigators blinded to patient information. In case of ambiguous results, the sample was analysed a second time.

### 2.6. Quantification of Serum 25(OH) Vitamin D Levels

A peripheral venous blood sample was taken from each patient at the time of inclusion. Serum levels of 25(OH) vitamin D were quantified by ELISA using commercial kits (My BioSource; MBS580159, San Diego, CA, USA). All procedures were performed according to the manufacturer's recommendations. All measurements were performed by the same researchers, who were blinded to the clinical characteristics of the patients to avoid measurement biases.

### 2.7. Statistical Analysis

Qualitative variables were expressed as frequencies and percentages. Quantitative variables are expressed as the median, minimum (min), and maximum (max).

We identified genotypic frequencies by direct counting. Allelic frequencies were determined by direct counting of the observed genotypic frequencies. The chi-square test (or Fisher's exact test if required) was used for the comparison of proportions. For variables that did not satisfy the assumptions of parametric tests, Mann–Whitney *U* nonparametric tests were performed.

Odds ratio (OR) and the 95% confidence interval (95% CI) were calculated for genetic characteristics. The Kruskal-Wallis test was performed to compare serum 25(OH) vitamin D levels between genotypes. We also performed post hoc tests with the Bonferroni correction for multiple comparisons.

Hardy-Weinberg equilibrium (HWE) for CYP2R1 rs10766197 and CYP27B1 rs10877012 polymorphisms was evaluated in the groups and was determined by comparing the observed and expected data using the chi-squared test.

We performed a multivariable logistic regression analysis to identify variables associated with the MS. This model was adjusted by confunders (age and gender) and genotypes for genes. For the *CYP2R1 gene* rs10766197 GG was stated as referent and GA + AA was considered as risk. For the *CYP27B1* gene rs10877012 GG was used as referent and GT + TT was selected as risk. The variables included in the model were selected using a stepwise method.

A *p* value was considered significant at the *p* ≤ 0.05 level. Data were analysed with R 4.1.0 base [28] and with the epiR [29] and ggplot2 packages [30].

### 2.8. Ethics and Consent

This study was approved by the Ethics Committee and Biosafety Committee from Centro Universitario de Ciencias de la Salud (CUCS) Universidad de Guadalajara, Jalisco, Mexico, with registration number CI-03519. This study was conducted according to the principles expressed in the Declaration of Helsinki. All study participants voluntarily provided written informed consent.

## 3. Results

We assessed 116 patients with MS and 226 individuals in the control group. The genotype distributions of the SNPs rs10766197 and rs10877012 in the control group were consistent with HWE (rs10766197 *p* > 0.05 and rs10877012 *p* > 0.05) (data not shown in tables).

The baseline characteristics of the MS patients are shown in Table 1. Of all MS patients included, 65.5% were females. We observed a median age of 38 (19-66) years and a median of 7.5 (1-28) years of disease evolution. Overall, 33.6% of the patients with MS presented severe disease progression (PI > 0.6), while 66.4% presented low progression of the disease (IP < 0.6). Deficiency of serum 25(OH) vitamin D levels in 73.3% of MS patients was observed. The most prescribed treatment in MS was glatiramer acetate (33.6%), followed by interferons (23.3%) and rituximab (10.3%).

**Figure 1:**
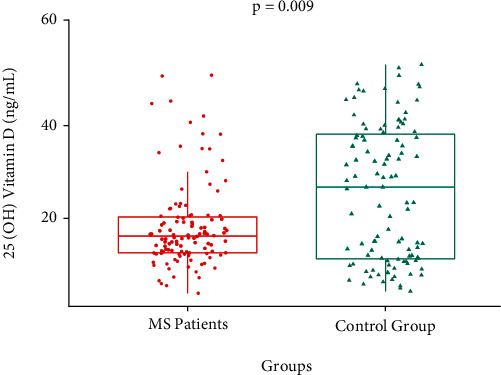
Levels of 25(OH) vitamin D in serum between MS patients and the control group (*p* = 0.009). The comparison was performed by Mann–Whitney *U* test.

Table 2 presents the comparison of sociodemographic, serological, and genetic characteristics between MS patients and the control group. For rs10766197 of the *CYP2R1* gene, we observed a higher frequency of the heterozygous genotype GA (50.0%) in MS patients, while in the control group, we observed an increased frequency of the wild-type homozygous genotype GG (49.6%); however, these differences were not statistically significant (*p* = 0.08). The A allele was observed at a higher frequency in MS patients than in the control group (37.9% vs. 30.5%, *p* = 0.05). For rs10877012 of the *CYP2B1* gene, we observed a similar frequency of genotypes in both groups. We did not identify a significant difference between MS patients and the control group (*p* = 0.90). Additionally, in a subgroup of 106 individuals in the control group, we compared the 25(OH) vitamin D levels. Higher levels of 25(OH) vitamin D were observed in the control group than in the MS group (*p* = 0.009) (Figure 1). We identified a higher percentage of individuals who had sufficient 25(OH) vitamin D levels in the control group (43.4%, *p* = 0.003) and a higher percentage of individuals deficient in vitamin D in MS patients (73.3%, *p* < 0.001).

Table 3 shows the comparison of clinical characteristics between severe progression versus low progression in MS patients. We observed the same proportion of females in the severe progression and low progression groups (64.1% vs. 65.1%, respectively). We observed a shorter disease duration in the severe progression group than in the low progression group (3 years vs. 12 years, *p* < 0.001). In quantitative serum 25(OH) vitamin D levels, we did not observe a significant difference between the severe and low progression groups. In a stratified comparison of serum 25 (OH) vitamin D levels, we observed a higher percentage of individuals with deficient levels (<20 ng/mL) and found a significant difference between groups (*p* = 0.037). No differences were observed in genotypes and allele frequencies of either polymorphism between patients with severe vs. low disease progression.

Table 4 compares the genotypic and allelic frequencies of polymorphisms rs10766197 and rs10877012 between MS patients and the control group. In a comparison of the GG, GA, and AA genotypes of the rs10766197 polymorphism of *CYP2R1* between MS patients and the control group, a trend to almost significant differences were observed (*p* = 0.08). In a comparison of the GG, GT, and TT genotypes of the rs10877012 polymorphism of *CYP2B1* between MS patients and the control group, no differences were observed. OR and their 95% CI were calculated for the comparison between dominant and recessive genetic models. We observed a high risk of MS in the dominant model of rs10766197 of the *CYP2R1* gene (OR = 1.67; 95%CI = 1.05 − 2.64; *p* = 0.02). No differences in the risk of other genetic models or in rs10877012 of the *CYP2B1* gene polymorphism were observed.

Distribution analysis of serum 25(OH) vitamin D levels according to the genotypes of polymorphisms stratified between MS patients and the control group revealed that patients who carried the genotype GG for rs10766197 presented higher levels, with a median of 17.44 ng/mL (ranges of 8.45-45.32 ng/mL).

In data not shown, we performed a multivariable logistic regression analysis. After adjusting by confounders variables, we found that age (OR = 0.95, CI 95% 0.92-0.98; *p* = 0.001) and gender ratio (OR = 2.18, CI 95% 1.16-4.2; *p* = 0.017) were associated with an increase of risk for MS. The GA + AA genotypes of *CYP2R1* rs10766197 polymorphism were associated with an increase of MS (OR = 1.87, CI 95% 1.07-3.29; *p* = 0.02). However, the *CYP27B1* rs10877012 polymorphism was not associated with the disease.

**Figure 2:**
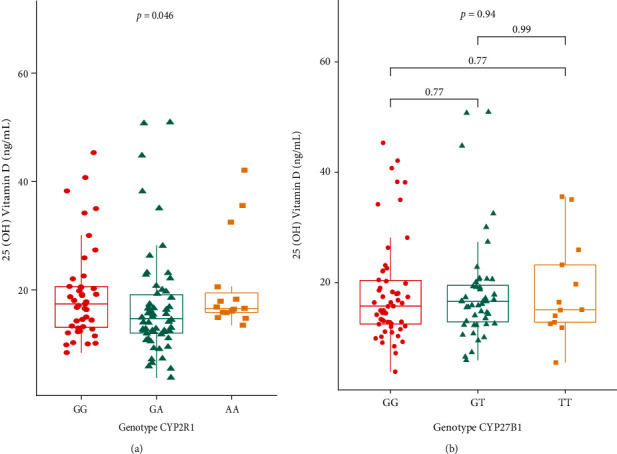
Comparison of levels of 25(OH) vitamin D in serum according to genotypes of single nucleotide polymorphisms rs10766197 of CYP2R1 gene and rs10877012 of CYP27B1 gene between MS patients. (a) Comparison of the levels of 25(OH) vitamin D in serum between genotypes of rs10766197 of the CYP2R1 gene. (b) Comparison of the levels of 25(OH) vitamin D in serum between genotypes of rs10877012 of the CYP27B1 gene. Data are presented as medians with ranges (min-max) of 25(OH) vitamin D (ng/mL). The comparison between genotype groups was designed by the Kruskal-Wallis test and Bonferroni correction to perform multiple comparisons.

In the comparison of serum 25(OH) vitamin D levels between GG, GA, and AA genotypes, we observed significant differences (*p* = 0.046), and in the post hoc analysis, a notably significant difference was observed between the GA and AA genotypes (*p* = 0.029). In the comparison of serum 25(OH) vitamin D levels between genotypes of rs10877012 of the *CYP2B1* gene, we did not observe a significant difference (*p* = 0.94) (Figure 2) (data not shown in tables).

In the comparison of genotypic and allelic frequencies of polymorphisms rs10766197 of the *CYP2R1* gene and rs10877012 of the *CYP27B1* gene between severe progression vs. low progression in MS patients, we observed a similar frequency of the AA genotype for rs10766197 of the *CYP2R1* gene (15.4% in severe progression vs. 11.6% in low progression). Furthermore, for rs10877012 of *CYP2B1* gene, we observed a higher frequency in of the heterozygous GT genotype in the severe group (48.4%) than low progression group, in which we observed the wild homozygous GG with high frequency (51.9%). No significant differences were observed in the comparison of genotypes between the severe and low MS progression groups (data not shown in tables).

## 4. Discussion

In this study, we observed that the rs10766197 polymorphism of the *CYP2R1* gene confers a risk of MS. However, the rs10877012 polymorphism of the *CYP27B1* gene was not associated with the risk of MS. We report the first allelic and genotypic frequencies in a Latin American country for the rs10766197 polymorphism of the *CYP2R1* gene and the rs10877012 polymorphism of the *CYP27B1* gene.

CYP2R1 is responsible for the hydroxylation of vitamin D to 25(OH) vitamin D in the first activation step. It is thought to be an important determinant of the vitamin D metabolic pathway, as it shows a high affinity for vitamin D [31]. The SNP rs10766197 is located in the promoter region of the *CYP2R1* gene, which causes differences in gene expression [32]. The association between the rs10766197 polymorphism of the *CYP2R1* gene and MS has been previously identified. Scazzone et al. (2017) reported that an association between the GA genotype (heterozygous minor allele carriers) in Italian patients was 46% in MS patients vs. 43% in controls (OR 2.19, 95% CI 1.09-4.39, *p* = 0.03), and the frequency of the AA genotype (homozygous minor allele carriers) was 39% in MS patients vs. 25% in controls (OR 3.18, 95% CI 1.52-6.65, *p* 5 0.002) [19]. Other studies analysed other polymorphisms in this gene. Laursen et al. found a significant association of CYP2R1 rs10741657 with MS risk in a cross-sectional study performed on the Danish population [33].

In our study, we found that the frequency of vitamin D deficiency was lower in MS patients than in controls. Our findings are supported by other studies; Oliveira et al., 2017 reported an association between serum levels of 25(OH) vitamin D and MS, reporting and media of MS patients (26.12 ± 8.47 ng/mL) vs. controls (29.71 ± 9.17 ng/mL, *p* = 0.02) [34], and Munger et al., 2006 found that the risk of MS decreased with increasing serum levels of 25(OH) vitamin D [35]. Several hypotheses have been proposed in advance to explain the role of vitamin D in the MS. First, vitamin D influences the balance of CD4 T lymphocytes, by decreasing Th1 and Th17 cell differentiation and promoting Th2 and regulatory T cells (Tregs) proliferation. In the development of MS, CD4 T lymphocytes (or T helper lymphocytes, including Th1, Th2, Th17 subsets) play a crucial role in the activation of myelin-specific Th1 and Th17 cells, which drives the inflammation within the central nervous system [36, 37]. Second, MS is characterized by progressive demyelination, vitamin D is deemed to have a role in the myelination and remyelination processes [38–41], and finally, 80% of MS-associated genes are enriched for VDREs in their promoter region and consequently, and they are regulated by vitamin D [42].

CYP27B1, encoding 1a-hydroxylase, which converts 25(OH) vitamin D to its active form, 1,25(OH)2D, is the cytochrome P450 gene most strongly associated with vitamin D status [15]. The SNP rs10877012 located on the 5′ untranslated region (UTR) may affect transcript stabilization and the posttranscriptional control of mRNA [43]. For the rs10877012 polymorphism of the *CYP27B1* gene, we observed that genotype or allele does not confer a clinically relevant risk for the development of MS. These results are in agreement with those reported by Simon et al. in American women with MS; in the study, findings do not support a role for an independent effect of the vitamin D-related gene polymorphisms investigated and the risk of MS [44]. The authors also identified three additional SNPs on the *CYP27B1* gene associated with MS [19].

Genome-wide association studies (GWAS) and other epidemiological studies have shown that SNPs within the *CYP2R1* gene modulate 25(OH) vitamin D levels [45–47]. In our study, we found that MS patients carrying one or two A alleles had lower 25(OH) vitamin D serum levels in comparison to patients carrying allele G. Scazzone et al. reported the rs10766197 distribution in MS patients, stratified according to the 25(OH) vitamin D concentrations, and the patients who carried the AA genotype presented a trend of lower levels of 25(OH) vitamin D in comparison to those with a genotype of GG or GA, although the difference was not statistically significant [14]. In contrast, other studies have reported an association of the rs10766197 polymorphism with the highest mean levels of 25(OH) vitamin D in the healthy Chinese population [48].

We did not find an association between the rs10877012 polymorphism of the *CYP27B1* gene and 25(OH) vitamin D levels. However, this association has been reported in other diseases. Ramos-Lopez et al. reported that the rs10877012 C allele was associated with lower levels of 25(OH) vitamin D in a study of patients with gestational diabetes [20].

There could be several reasons why we were not able to find an association between the SNPs and 25(OH) vitamin D levels. These inconsistent results could be derived from differences in population selection and ethnicity. The serum 25(OH) vitamin D levels of the population we studied were much lower than those in other published reports [49–51]. ENSANUT in 2006 reported that approximately 30% of adults aged 20 to 60 years have hypovitaminosis D [51].

It has been reported in Mexico that 37% of women of reproductive age have deficient levels and 50% have insufficient levels of vitamin D [52].

To date, to the best of our knowledge, this is the first study to analyse the association between these two polymorphisms, 25(OH) vitamin D levels and MS, in Mexican patients, which enabled us to examine the interactions between the *CYP2R1* gene, *CYPB27* gene, and vitamin D levels to assess how these synergisms may influence MS risk.

There are limitations to the present investigation. First, in relation to the findings of the main effects of individual SNPs and MS risk, these genes were not submitted to an exhaustive examination. However, we cannot exclude the possibility that other gene regions may be important. Second, we did not measure 25-hydroxylase activity. Third, our study was limited to a small sample size.

MS is a multifactorial disease, and this finding supports the notion that risk factors may be relevant only in a proportion of the population with underlying genetic susceptibility [53]. Future investigations that could replicate the findings in this work are necessary to verify the biological precept of the plausibility of gene-environment interactions as they relate to vitamin D and MS risk.

## 5. Conclusion

Reduced serum 25(OH) vitamin D levels were observed in MS patients, although these levels were not associated with disease progression. Carriers of GA + AA rs10766197 of the *CYP2R1* gene had an increased risk of MS. None of these polymorphisms was associated with severe progression on MS disease.

## Figures and Tables

**Table 1:** Sociodemographic and clinical descriptive characteristics in multiple sclerosis patients.

Variable	Multiple sclerosis *n* = 116
Female, *n* (%)	76 (65.5)
Age, (years)	38 (19-66)
Disease characteristics	
Disease evolution, (years)	7.5 (1.0-28.0)
EDSS, (score)	3.0 (0.0-7.0)
Progression index, (score)	0.38 (0.0-4.5)
Severe progression (IP > 0.6), *n* (%)	39 (33.6)
Low progression (IP ≤ 0.6), *n* (%)	77 (66.4)
Serum 25(OH) vitamin D levels, (ng/mL)	18.3 (3.8-50.9)
Sufficient (>30 ng/mL), *n* (%)	14 (12.0)
Insufficient (20-30 ng/mL), *n* (%)	17 (14.7)
Deficient (<20 ng/mL), *n* (%)	85 (73.3)
Treatment	
Glatiramer acetate, *n* (%)	39 (33.6)
Interferon, *n* (%)	27 (23.3)
Rituximab, *n* (%)	12 (10.3)
Fingolimod, *n* (%)	11 (9.5)
Dimethyl fumarate, *n* (%)	7 (6.0)
Azathioprine, *n* (%)	5 (4.3)
Natalizumab, *n* (%)	4 (3.5)
No treatment, *n* (%)	11 (9.5)

EDSS: Expanded Disability Status Scale of Kurtzke for Multiple Sclerosis; IP: index progression (EDSS/disease evolution). Qualitative variables were expressed as frequency and percentage. Quantitative variables were expressed as median and min-max.

**Table 2:** Comparison of sociodemographic, serological, and genetic characteristics of multiple sclerosis patients versus control group.

Variable	Multiple sclerosis *n* = 116	Control group *n* = 226	*p*
Female, *n* (%)	76 (65.5)	185 (81.9)	0.001
Age (years)	38 (19-66)	46.0 (21-66)	<0.001
Serum levels			
25(OH) vitamin D levels (ng/mL), *n* (%)	16.2 (3.8-50.9)	26.8 (4.3 − 53.2)∗	0.009
Sufficient (>30 ng/mL), *n* (%)	14 (12.0)	46 (43.4)∗	
Insufficient (20-30 ng/mL), *n* (%)	17 (14.7)	14 (13.2)∗	0.009
Deficient (<20 ng/mL), *n* (%)	85 (73.3)	46 (43.4)∗	
Genetic characteristics			
rs10766197, *CYP2R1 gene*			
Genotypes			
GG, *n* (%)	43 (37.1)	112 (49.6)	
GA, *n* (%)	58 (50.0)	90 (39.8)	0.08
AA, *n* (%)	15 (12.9)	24 (10.6)	
Allele 2*n* = 684	2*n* = 232	2*n* = 452	
A, *n* (%)	88 (37.9)	138 (30.5)	0.05
G, *n* (%)	144 (62.1)	314 (69.5)	0.05
rs10877012, *CYP2B1 gene*			
Genotypes			
GG, *n* (%)	55 (47.4)	101 (44.7)	
GT, *n* (%)	48 (41.4)	98 (43.4)	0.90
TT, *n* (%)	13 (11.2)	27 (11.9)	
Allele 2*n* = 684	2*n* = 232	2*n* = 452	
G, *n* (%)	158 (68.1)	300 (66.4)	0.65
T, *n* (%)	74 (31.9)	152 (33.6)	0.65

For rs10766197 of *CYP2R1 gene*: GG: wild homozygous; GA: heterozygous; AA: polymorphic homozygous. For rs10877012 of *CYP2B1 gene*: GG: wild homozygous; GT: heterozygous; TT: polymorphic homozygous. Qualitative variables were expressed as frequency and percentage. Quantitative variables were expressed as median and min-max. Comparisons between medians were performed using Mann–Whitney *U* test. Comparison between proportions was performed using Chi-square test (or Fisher exact test if applicable). *p* values were obtained comparing multiple sclerosis versus control group. ∗Serum levels of 25(OH) vitamin D were determined in 107 healthy controls.

**Table 3:** Comparison of clinical and genetic characteristics between severe progression and low progression in multiple sclerosis patients.

Variable	Severe progression (IP > 0.6) *n* = 39	Low progression (IP ≤ 0.6) *n* = 77	*p*
Female, *n* (%)	25 (64.1)	51 (66.2)	0.98
Age, (years)	36 (19-59)	40 (19-66)	0.13
Disease characteristics			
Disease evolution, (years)	3 (1-9)	12 (2-28)	<0.001
EDSS, (score)	4.0 (1-7)	2.5 (0-7)	0.008
Serum 25(OH) vitamin D levels, (ng/mL)	15.7 (3.8-50.9)	16.4 (5.4-45.3)	0.88
Sufficient (>30 ng/mL), *n* (%)	4 (10.3)	13 (16.9)	
Insufficient (20-30 ng/mL), *n* (%)	12 (30.7)	9 (11.7)	0.037
Deficient (<20 ng/mL), *n* (%)	23 (59.0)	55 (71.4)	
Treatment			
Glatiramer acetate, *n* (%)	9 (23.1)	30 (39.0)	0.10
Interferon, *n* (%)	7 (17.9)	20 (25.9)	0.36
Rituximab, *n* (%)	9 (23.1)	3 (3.9)	0.003
Fingolimod, *n* (%)	3 (7.7)	8 (10.4)	0.75
Dimethyl fumarate, *n* (%)	4 (10.3)	3 (3.9)	0.22
Azathioprine, *n* (%)	3 (7.7)	2 (2.6)	0.33
Natalizumab, *n* (%)	1 (2.5)	3 (3.9)	1.00
No treatment, *n* (%)	3 (7.7)	8 (10.4)	0.49
Genetic characteristics			
rs10766197, *CYP2R1 gene*			
Genotypes			
GG, *n* (%)	12 (30.8)	31 (40.3)	
GA, *n* (%)	21 (53.8)	37 (48.0)	0.58
AA, *n* (%)	6 (15.4)	9 (11.7)	
Allele 2*n* = 232	2*n* = 78	2*n* = 154	
A, *n* (%)	33 (42.3)	55 (35.7)	0.33
G, *n* (%)	45 (57.7)	99 (64.3)	0.33
rs10877012, *CYP2B1 gene*			
Genotypes			
GG, *n* (%)	15 (38.5)	40 (51.9)	
GT, *n* (%)	19 (48.7)	29 (37.7)	0.39
TT, *n* (%)	5 (12.8)	8 (10.4)	
Allele 2*n* = 232	2*n* = 78	2*n* = 154	
T, *n* (%)	49 (62.8)	45 (29.2)	<0.001
G, *n* (%)	29 (37.2)	109 (70.8)	<0.001

EDSS: Expanded Disability Status Scale of Kurtzke for Multiple Sclerosis; IP: index progression (EDSS/disease evolution). For rs10766197 of *CYP2R1 gene*: GG: wild homozygous; GA: heterozygous; AA: polymorphic homozygous. For rs10877012 of *CYP2B1 gene*: GG: wild homozygous; GT: heterozygous; TT: polymorphic homozygous. Qualitative variables were expressed as frequency and percentage. Quantitative variables were expressed as median and min-max. Comparisons between medians were performed using Mann–Whitney *U* test. Comparison between proportions was performed using Chi-square test (or Fisher exact test if applicable). *p* values were obtained comparing multiple sclerosis with severe progression versus multiple sclerosis with low progression.

**Table 4:** Comparison of genotypic and allelic frequencies of polymorphisms rs10766197 of *CYP2R1 gene* and rs10877012 of *CYP2B1 gene*, between multiple sclerosis patients and control group.

	Multiple sclerosis *n* = 116	Control group *n* = 226	OR	95% CI	*p*
rs10766197, *CYP2R1 gene*					
*Genotypes*					
GG *n* = 155 (%)	43 (37.1)	112 (49.6)	—	—	
GA *n* = 140 (%)	58 (50.0)	90 (39.8)	—	—	0.08
AA *n* = 39 (%)	15 (12.9)	24 (10.6)	—	—	
GA + AA versus GG (as referent)	73 (62.9)	114 (50.4)	1.67	1.05-2.64	0.03
GA + GG versus AA (as referent)	101 (87.0)	202 (89.4)	0.8	0.40-1.59	0.59
*Alleles 2n =684*	*2n =232*	*2n =452*			
A allele, 2n =226 (%)	88 (37.9)	138 (30.5)	1.39	0.99-1.94	0.05
G allele, 2*n* = 458 (%)	144 (62.1)	314 (69.5)	0.72	0.52-1.00	0.05
rs10877012, *CYP2B1 gene*					
Genotypes					
GG *n* = 156 (%)	55 (47.4)	101 (44.7)	—	—	
GT *n* = 146 (%)	48 (41.4)	98 (43.4)	—	—	0.90
TT *n* = 40 (%)	13 (11.2)	27 (11.9)	—	—	
GT + TT versus GG (as referent)	61 (52.6)	125 (55.3)	0.89	0.57-1.40	0.63
GG + GT versus TT (as referent)	103 (88.8)	199 (88.1)	1.08	0.53-2.17	0.84
Allele 2*n* = 684	2*n* = 232	2*n* = 452			
G allele, 2*n* = 458 (%)	158 (68.1)	300 (66.4)	1.08	0.77-1.52	0.65
T allele, 2*n* = 226 (%)	74 (31.9)	152 (33.6)	0.92	0.66-1.30	0.65

For rs10766197 of *CYP2R1 gene*: GG: wild homozygous; GA: heterozygous; AA: polymorphic homozygous; for rs10877012 of *CYP2B1* gene: GG: wild homozygous; GT: heterozygous; TT: polymorphic homozygous. OR: odds ratio risk; 95% CI: 95% confidence interval. *p* values were obtained comparing multiple sclerosis versus control group.

## Data Availability

The database used to support the findings of this study is available on request. If this database is required, please direct the correspondence to Dr. Ana M. Saldaña-Cruz (ana.saldanac@academicos.udg.mx).
